# Assigning protein function from domain-function associations using DomFun

**DOI:** 10.1186/s12859-022-04565-6

**Published:** 2022-01-15

**Authors:** Elena Rojano, Fernando M. Jabato, James R. Perkins, José Córdoba-Caballero, Federico García-Criado, Ian Sillitoe, Christine Orengo, Juan A. G. Ranea, Pedro Seoane-Zonjic

**Affiliations:** 1 Department of Molecular Biology and Biochemistry, University of Malaga, Bulevar Louis Pasteur, 31, 29010 Malaga, Spain; 2 CIBER of Rare Diseases, Av. Monforte de Lemos, 3-5. Pabellon 11. Planta 0, 28029 Madrid, Spain; 3 Department of Structural and Molecular Biology, University College London, Gower Street, London, WC1E 6BT UK; 4 Institute of Biomedical Research in Malaga (IBIMA), Dr. Miguel Díaz Recio, 28, 29010 Malaga, Spain

**Keywords:** Function prediction, CATH, DomFun, Protein domains, CAFA

## Abstract

**Background:**

Protein function prediction remains a key challenge. Domain composition affects protein function. Here we present DomFun, a Ruby gem that uses associations between protein domains and functions, calculated using multiple indices based on tripartite network analysis. These domain-function associations are combined at the protein level, to generate protein-function predictions.

**Results:**

We analysed 16 tripartite networks connecting homologous superfamily and FunFam domains from CATH-Gene3D with functional annotations from the three Gene Ontology (GO) sub-ontologies, KEGG, and Reactome. We validated the results using the CAFA 3 benchmark platform for GO annotation, finding that out of the multiple association metrics and domain datasets tested, Simpson index for FunFam domain-function associations combined with Stouffer’s method leads to the best performance in almost all scenarios. We also found that using FunFams led to better performance than superfamilies, and better results were found for GO molecular function compared to GO biological process terms. DomFun performed as well as the highest-performing method in certain CAFA 3 evaluation procedures in terms of $F_{max}$ and $S_{min}$ We also implemented our own benchmark procedure, Pathway Prediction Performance (PPP), which can be used to validate function prediction for additional annotations sources, such as KEGG and Reactome. Using PPP, we found similar results to those found with CAFA 3 for GO, moreover we found good performance for the other annotation sources. As with CAFA 3, Simpson index with Stouffer’s method led to the top performance in almost all scenarios.

**Conclusions:**

DomFun shows competitive performance with other methods evaluated in CAFA 3 when predicting proteins function with GO, although results vary depending on the evaluation procedure. Through our own benchmark procedure, PPP, we have shown it can also make accurate predictions for KEGG and Reactome. It performs best when using FunFams, combining Simpson index derived domain-function associations using Stouffer’s method. The tool has been implemented so that it can be easily adapted to incorporate other protein features, such as domain data from other sources, amino acid k-mers and motifs. The DomFun Ruby gem is available from https://rubygems.org/gems/DomFun. Code maintained at https://github.com/ElenaRojano/DomFun. Validation procedure scripts can be found at https://github.com/ElenaRojano/DomFun_project.

**Supplementary Information:**

The online version contains supplementary material available at 10.1186/s12859-022-04565-6.

## Background

Determining protein function is one of the major goals of bioinformatics. A key factor influencing the role of a given protein is its domain composition [1, 2]. Although domains can have distinct functions when examined individually, their combination within a given protein is what gives rise to its overall role in cellular processes [3]. As such, we must first understand individual domains and then investigate how they contribute to protein function. Approaches like dcGO use information from resources such as the Gene Ontology (GO) [4, 5], to statistically infer domain annotation [6].

Various features are important for function prediction, including sequence homology and conserved structure, which are used to classify protein domains by resources such CATH-Gene3D [7, 8], which uses a hierarchical classification system with the most specific group being the homologous superfamily [9].

Within the homologous superfamilies, domains can be further sub-divided into functional families (FunFams), based on shared patterns of sequence conservation [10]. Such domain families have been used by algorithms such as FunFHMMer to predict function at the protein level [9]. Furthermore, FunFam domain information has been used to predict protein functional sites using machine learning [11].

In recent years, we have developed tools to analyse and extract information from network-based data structures [12, 13]. We used them to find associations between pathological phenotypes and genomic mutations [14–17], and predict the genes involved in the development of rare diseases [18]. Nevertheless, without adequate functional knowledge of the proteins encoded by these genes, we cannot fully understand the underlying mechanisms leading to disease.

**Fig. 1:**
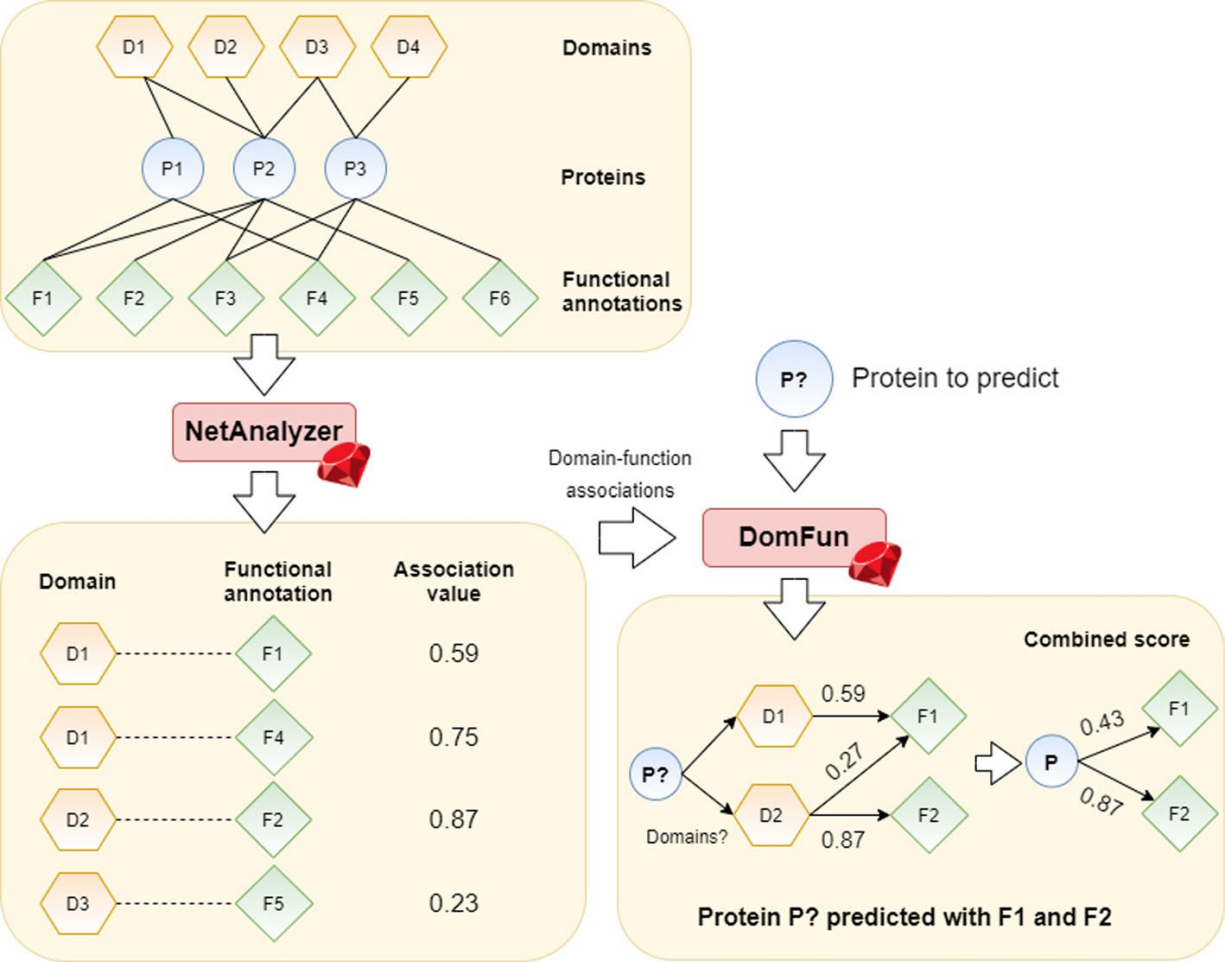
Workflow of the procedure followed in this study. We first built the domain-protein-function tripartite network. Then, we calculated associations between domains and functional annotations (through shared proteins) with NetAnalyzer. Once calculated, we combine these domain-function associations to predict proteins function with DomFun. For a given protein, DomFun obtains its constituent domains and their associated functions. These domain-function association values are combined to obtain protein-function scores

Here, we present DomFun, a framework that uses associations between protein domains and functional annotation to predict protein function (Fig. 1).

These associations are calculated by exploiting tripartite networks connecting domains and functional groups via proteins [19], using GO terms and pathways from KEGG [20] and Reactome [21]. For a given protein, DomFun obtains its constituent domains and their functional associations. The association scores are then combined to predict functional annotation.

We validated our method using the prediction benchmark of the third version of the Critical Assessment of Functional Annotation challenge (CAFA 3) [22]. This evaluation method is widely used to evaluate protein function prediction methods. We focussed on GO annotation for proteins from multiple organisms in the three GO sub-ontologies: molecular function (GOMF), biological process (GOBP) and cellular component (GOCC). We have also developed and applied our own benchmarking protocol, named Pathway Prediction Performance (PPP).

DomFun can be used to predict protein function for multiple organisms for which protein domain and functional annotation information is available. Although we used CATH-Gene3D annotation in this work, other features can be used. It can be downloaded from https://rubygems.org/gems/DomFun. Ruby code is available from https://github.com/ElenaRojano/DomFun and the workflow from https://github.com/ElenaRojano/DomFun_project. Technical information to install and use DomFun is provided in these repositories.

## Implementation

We have developed and implemented DomFun, a tool to predict function for a given protein based on associations between its constituent domains and functions, obtained from various annotation-databases. Associations are calculated using a tripartite network comprised of domains, proteins and functional annotation. The DomFun algorithm works in the following manner: Based on a training dataset, protein-domain and protein-function annotation data are combined to produce a tripartite network of 3 layers: domains-proteins-functions. This tripartite network is analysed using the NetAnalyzer software [12]. NetAnalyzer take as input a multipartite network and calculates associations between different layers within this network. In the context of this work, the layers are: domains, proteins and functions, and the associations are calculated between the domains and functions layers, bases on the connections via proteins. The output is therefore a list of pairs of domains and functions, with corresponding association values. Lists, with their corresponding association values, were calculated for 4 different association indices, mathematical details of which are described in the next section.

Then, given a testing protein, DomFun predicts function for this protein by obtaining all of its annotated domains and searching for their associated functions in the list generated by NetAnalyzer. The scores for these associated functions are then combined using the methods whose mathematical formulae are described below, to obtain overall scores, which represent the predicted functions for the protein.

An overview of the architecture of the software implementation is described in Fig. 1.

### Protein, domain and annotation data sources

To build the domain-protein-function tripartite networks used to calculate the domain-function associations with DomFun, two types of relations were combined: protein-function and protein-domain.

The workflow followed to perform the different steps explained next and data download links are included in a GitHub repository at https://github.com/ElenaRojano/DomFun_workflow.

#### Protein-function annotation

We used two datasets to establish protein-function relations.

The first dataset was based on the functional annotation included in the third Critical Assessment of Functional Annotation challenge (CAFA 3) [22]. We used 66,841 protein identifiers available for the CAFA 3 training set (35,086 annotated in GOMF, 50,813 in GOBP and 49,328 in GOCC) to construct the protein-annotation layer for six tripartite networks. This information was downloaded from CAFA 3 repository at https://www.biofunctionprediction.org/cafa-targets/CAFA3_training_data.tgz.

The second dataset was downloaded from UniProt (release 2021_02). We obtained 23,391,902 proteins both manually curated (Swiss-Prot) and computationally inferred (TrEMBL) for multiple species. We also downloaded their annotations in GOMF, GOBP, GOCC, KEGG and Reactome. We discarded proteins with GO annotation tagged as Inferred from Electronic Annotation (IEA) to ensure high-quality annotations for our study. We also discarded all protein tagged as *fragments* and protein fusions. We used this information to construct the protein-annotation layer for ten different tripartite networks. After these filters, we were left with 4,283,876 annotated proteins. Please note that the same protein may have annotation in any of the GO sub-ontologies, KEGG or Reactome.

#### Protein-domain annotation

To establish protein-domain relations, we used protein domains classified into homologous superfamilies and FunFams from the protein structure classification database CATH-Gene3D [8]. Superfamily classification is performed by grouping sequences likely to have an evolutionary relationship [9]. FunFams are a sub-classification of superfamilies based on shared patterns of sequence conservation related to function determining residues [23]. For this analysis, we used the CATH-Gene3D release v4_3_0, including 1,307,795 proteins from 1705 species, 4245 different superfamilies and 171,425 FunFams.

### Tripartite network construction and association index calculation

We obtained multiple sets of protein-function and protein-domain relations. In total we built 16 networks connecting domains to functional annotation via shared proteins, one for each combination of protein-domain and protein-function datasets.

These domain-protein-function networks were analysed with NetAnalyzer, a tool that calculates associations in multipartite networks [12]. We employed this tool to calculate domain-function associations using the Jaccard similarity index (eq. 1), Simpson index (eq. 2), Pearson correlation coefficient (PCC) (eq. 3) and the hypergeometric index (HyI) (eq. 4), as described in [24].

1 $$\begin{aligned} Jaccard(D,F)= & {} \frac{|N_p(D) \cap N_p(F)|}{|N_p(D) \cup N_p(F)|} \end{aligned}$$

2 $$\begin{aligned} Simpson(D,F)= & {} \frac{|N_p(D)\cap N_p(F)|}{min(|N_p(D)|, |N_p(F)|)} \end{aligned}$$

3 $$\begin{aligned} PCC(D,F)= & {} \frac{|N_p(D)\cap N_p(F)|\cdot n_{T} - |N_p(D)\cdot N_p(F)|}{\sqrt{|N_p(D)|\cdot |N_p(F)|\cdot (n_{T} - |N_p(D)|)\cdot (n_{T} - |N_p(F)|)}} \end{aligned}$$

4 $$\begin{aligned} HyI(D,F)= & {} -log \sum \limits _{i = |N_p(D) \bigcap N_p(F)|}^{min(|N_p(D)|,|N_p(F)|)}\frac{{|N_p(D)|\atopwithdelims ()i}\cdot {n_{T} - |N_p(D)|\atopwithdelims ()|N_p(F)| - i}}{{n_{T} \atopwithdelims ()|N_p(F)|}} \end{aligned}$$

where $N_p(D)$ and $N_p(F)$ are the set of protein nodes connected to a given domain node D and a function node F, respectively, and $n_{T}$ is the total number of protein nodes in the network.

### Protein function prediction based on domain-function associations

The domain-function associations calculated using the above methods were used to predict protein function using DomFun. First, for a given protein, associated with a specific UniProt identifier, DomFun searches for its constituent domains within CATH-Gene3D. If domains are found, it then searches for any functions associated with them. If a protein contains multiple domains associated with the same functional annotation (Fig.  1), DomFun integrates the association values into a single combined score. This leads to a list of possible functions for the protein, ranked based on the strength of the domain-function associations, as described in the next section.

By calculating this score for all functions associated with at least one domain for a given protein, we obtain a vector of scores, which represents the predicted functions for the protein.

DomFun outputs a table of predicted functions for each protein, containing the UniProt identifier, the domains for that protein classified according to CATH-Gene3D superfamilies or FunFams, the predicted functions (GOMF, GOBP, GOCC, KEGG and Reactome) and the combined score for each putative protein-function association.

#### Combining domain-function association values

As mentioned above, if a protein contains multiple domains with the same function, these scores are combined into a single value. In the case of HyI values, these must be transformed into *P*-values by calculating their antilogarithm (base=10), which represents the probability of having an equal or greater number of interactions between a pair of nodes (i.e., proteins connecting domains and functional annotations) than would be expected by chance [24]. To integrate these *P*-values we use the Fisher’s combined probability test (eq. 5).

5 $$\begin{aligned} X^j = -2 \sum _{i=1}^{k} ln(p_{i}^j) \sim {\chi }^{2}_{2k} \end{aligned}$$

Where $p_{i}^j$ is the HyI-derived *P*-value for the number of interactions between a function *j* and the domain i, and 2*k* represents the degrees of freedom. *k* represents the total number of domains for each predicted protein. This formula gives the test statistic, from which the combined *P*-value can be derived, based on the $\chi ^{2}$ distribution and degrees of freedom.

When combining association values produced using PCC, Jaccard or Simpson index, Stouffer’s method was used to obtain, for each of the three metrics, combined association values between proteins and functions (eq. 6) [25]. For this, the association values are first converted to *Z*-scores and then combined using the following formula:

6 $$\begin{aligned} Z^j = \frac{ \sum _{i=1}^{k} Z_{i}^j}{\sqrt{k}} \sim Z \end{aligned}$$

For a given protein, an overall *Z*-score was calculated for each of the functions *j* associated with at least one domain *i* within this protein. This was calculated by summing the $Z_{i}^j$ scores for the domains in the protein associated with the given function and dividing by the square root of k—the number of domains in the protein associated with the given function.

The $Z_{i}^j$ scores were calculated for each given domain-function association value, by subtracting the mean association value for all domain-function associations, and dividing by the standard deviation.

7 $$\begin{aligned} Z_{i}^j={a_{i}^{j}-{{\bar{a}}} \over s} \end{aligned}$$

Where *a* represents the association values for all calculated domain-function associations; $a_{i}^{j}$ represents the association value for the domain *i* with a given function *j*; ${{\bar{a}}}$ represents the mean association value for all domain-function associations; and *s* represents the standard deviation for the values in *a*.

### DomFun evaluation methods

We evaluated the ability of DomFun to predict protein function using the CAFA 3 prediction benchmark for GOBP, GOMF and GOCC, using the methodology described in [26] with data available from the CAFA 3 website (https://www.biofunctionprediction.org/cafa). We also developed our own validation procedure to evaluate DomFun in terms of predicting function for KEGG and Reactome pathways. We refer to this procedure as Pathway Prediction Performance (PPP). We also used PPP for GOMF, GOBP and GOCC annotations — as similar evaluation values for the three GO sub-ontologies to those found with CAFA 3 would suggest that our benchmark procedure is reliable and lend confidence to the interpretation of the PPP results for KEGG and Reactome.

For this validation, we looked at the maximum value of the harmonic mean ($F_{max}$) of precision and recall (PR), and $S_{min}$, based on the semantic distance between predictions and known annotations, in line with CAFA 3.

In total, we made eight separate sets of predictions, for the four different association metrics, separately for FunFams and superfamilies. Rather than compare all eight sets against all methods benchmarked within the CAFA 3 results (146 distinct methods), we initially compared the eight sets to each other, to see which performed best across all testing scenarios. The top performing method was then compared against the best performing method from CAFA 3 for each scenario.

#### CAFA 3 prediction benchmark

We used data from CAFA 3, a challenge aimed at comparing various methods for predicting protein function, to evaluate the predictive capability of DomFun [22]. In brief, the idea was that competitors would predict annotation for a number of proteins, and then compare their predictions to experimentally determined functional annotation obtained during a given time-period ($t_{0}$-$t_{1}$). As a result, they acquired and published a dataset including a list of the proteins that obtained annotation during this time period, their annotations at the start of the challenge ($t_{0}$) and at the end of the challenge ($t_{1}$), and a benchmarking procedure with scripts to implement it.

The CAFA 3 dataset includes various sub-divisions to evaluate the prediction methods, as explained in [22, 26], including two modes of evaluation: full and partial, and two types of annotation: no knowledge and limited knowledge. Full evaluation penalizes models if they cannot predict for all GO sub-ontology terms; partial mode evaluates without this penalization.

With respect to the different proteins in the benchmark testing set, no knowledge proteins are those with no experimentally verified annotation in any of the three GO sub-ontologies at time t$_{0}$, but that accumulate at least one verified GO term between t$_{0}$ and t$_{1}$. Limited knowledge includes proteins with annotation in at least one GO sub-ontology, but not in all three at t$_{0}$ [22, 26]. We used different combinations to see with which one of our methods performed best in different scenarios.

We used the CAFA protein-centric evaluation mode. It calculates maximum F-measure $F_{max}$, using PR values for the proteins for which predictions could be made, to ascertain the performance of the predictive method. It also calculates the minimum semantic distance ($S_{min}$) between two GO terms (one from prediction and the other from the ground truth, i.e. CAFA 3). In addition, it calculates coverage, defined in this work as the fraction of benchmark proteins for which DomFun could make predictions. Formulae of these evaluation metrics are described in [26].

The CAFA 3 benchmark files include 3089 proteins. We consider this set as our testing set. We predicted GOMF, GOBP and GOCC for 2483 proteins from this set. As occurred with the training proteins set, we lost 606 testing proteins as they had no CATH domains. This loss affects prediction performance in terms of coverage.

We compared DomFun performance against the two baseline models, Naïve and BLAST. We generated both models following instructions provided by CAFA authors [22]. We also compared the performance of our methods with the top scoring methods from CAFA 3, in terms of both $F_{max}$ and $S_{min}$, for all three ontologies and all four combinations of evaluation type and mode. Analyses were made using all organisms.

According to CAFA submission rules, prediction scores must be within the (0.00,1.00] range. Therefore, we normalized the combined scores for each protein. In the case of scores calculated using Stouffer’s method, as PCC values were within a range of [-1.00,1.00], their absolute values were calculated before they were combined. For Jaccard and Simpson combined scores, normalization was performed as follows: combined scores were transformed to *Z*-scores and any value with absolute value greater than 2 was set to 2. Finally, the distribution was normalised to the (0.00,1.00] range by dividing each value by the distribution range (4) and adding 0.5. In the case of scores calculated using Fisher’s method, i.e. the combined HyI derived *P*-values, we transformed them into the (0.00,1.00] range by subtracting the *P*-value generated by the method from 1. To avoid spurious predictions, we removed predictions with a transformed score lower than 0.001.

To compare the results of the different association/combination methods used by DomFun on the CAFA 3 testing proteins, for both FunFams and superfamilies, we compared their results in terms of $F_{max}$ and $S_{min}$ using the Friedman test. If this gave a significant result ($p \le 0.05$), meaning there is a difference between groups, this was followed by post-hoc pair-wise Dunn’s tests to identify a significant difference between the top ranked method and the other methods ($p \le 0.05$) [27].

#### Pathway prediction performance

In the pathway prediction performance (PPP) validation procedure, we predicted protein function (across all three GO sub-ontologies, KEGG and Reactome) for all proteins that were used to build the domain-protein-function tripartite networks. We compared these predicted associations to the original protein-function associations, and built precision-recall (PR) curves. For this, all predicted annotations that matched the original annotation were considered true positives; all predictions that did not match the original annotation were considered false positives. This additional validation procedure was necessary to evaluate the results obtained using KEGG and Reactome. As we had already validated the GO annotation using the CAFA 3 benchmarking procedure, we were able to use the PPP procedure to compare the results using KEGG and Reactome to the GO results and put them in the context of the CAFA 3 predictions. PR curves were generated using the ROCR package [28].

## Results

We associated protein domains with functions by applying various metrics to tripartite networks formed by combining protein-domain and protein-function annotation via shared proteins. Functional annotation was obtained from the Gene Ontology molecular function (GOMF), biological process (GOBP) and cellular component (GOCC) sub-ontologies and KEGG and Reactome pathways.

The domain-function associations were then used by DomFun for protein function prediction. Two validation procedures were used, one using the training and testing proteins from the CAFA 3 benchmark dataset [22], and another based on all Uniprot proteins (Pathway Prediction Performance, PPP).

### CAFA 3 benchmark results

The CAFA 3 training set contains 98,567 proteins from multiple organisms, of which 50,813 had annotations in GOBP, 35,086 in GOMF and 49,328 in GOCC [22]. We used all proteins from these sets that had CATH-Gene3D annotation to build domain-protein-function tripartite networks for each of the sub-ontologies. In total, there were 41,453 proteins with domain and GOBP annotation, 30,650 with GOMF and 39,144 with GOCC, corresponding to a loss of 18.42%, 12.64% and 20.64% proteins, respectively, from the training set.

We used the domain-function associations calculated for the CAFA 3 training proteins at $t_{0}$ to predict GO annotation for the 3,089 testing proteins included in CAFA 3, of which 2,483 had domain annotation. Predictions were compared to the annotation obtained for these proteins between $t_{0}$ and $t_{1}$.

**Fig. 2:**
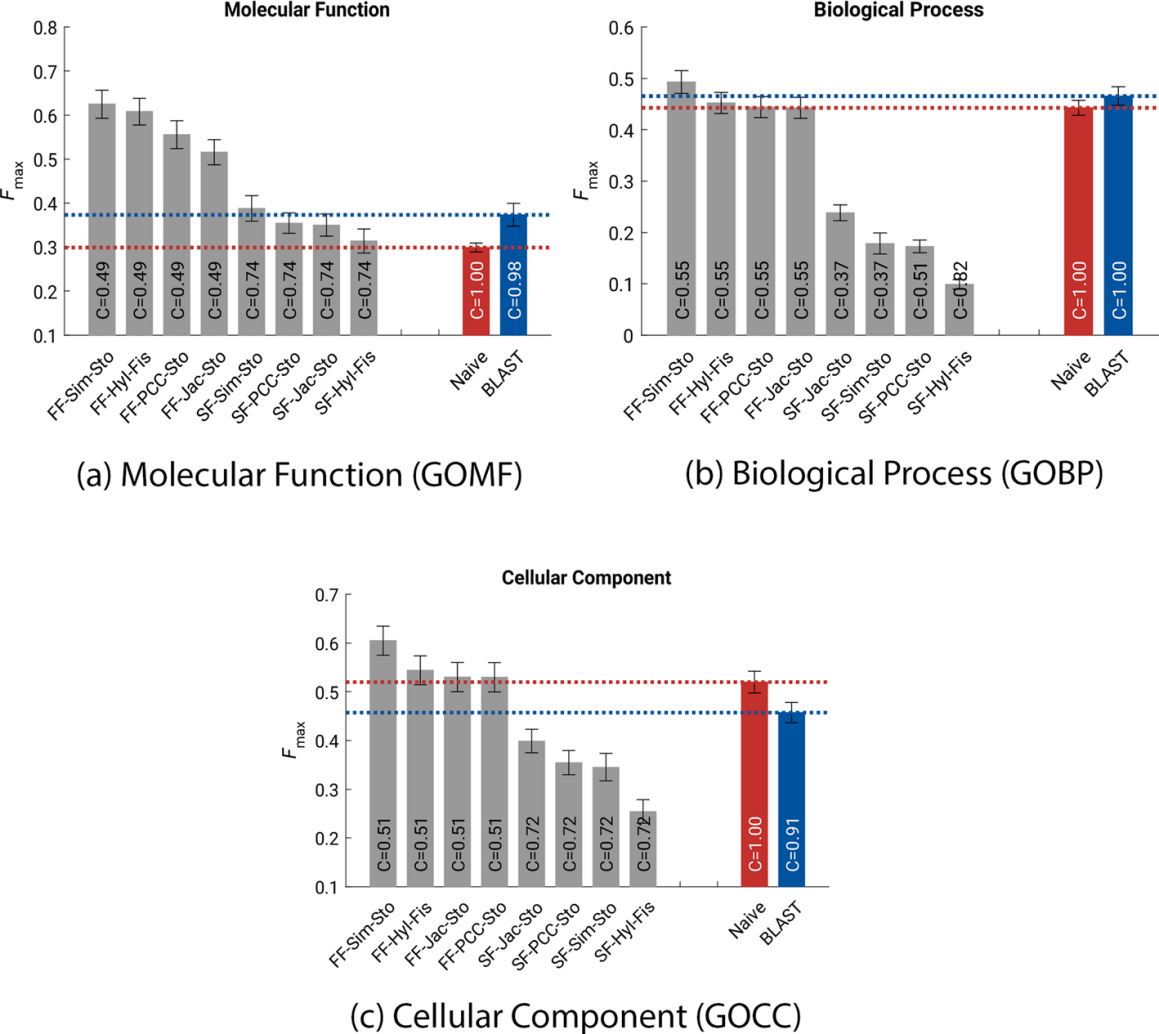
DomFun evaluation in terms of maximum *F***-measure** ($F_{max}$) calculation when predicting for Gene Ontology (GO) molecular function (GOMF) (a), biological process (GOBP) (b) and cellular component (c) (GOCC) terms using the CAFA 3 prediction benchmark. The associations between GO terms and protein domains, classified using FunFams (FF) and superfamilies (SF) separately, were calculated using four different association indices: Jaccard (Jac), Simpson (Sim), Pearson correlation coefficient (PCC) and hypergeometric index (HyI), and combined using either Fisher’s method (Fis) or Stouffer’s method (Sto). Results for the baseline methods BLAST and Naïve are also included for comparison. Coverage (*C*) values for each method are included within the bars. The CAFA 3 evaluation procedure was set to partial mode and limited knowledge

We analysed the results to evaluate which combination of domain family, association and integration methods led to the most accurate predictions. This was performed separately for the FunFam and homologous superfamily classifications. We initially compared the results of each method to each other and to the two CAFA 3 baseline methods (BLAST and Naïve) in terms of maximum F-measure $F_{max}$ (Fig. 2). The exact values are shown in Table 1 for the CAFA 3 limited-knowledge, partial evaluation procedure. Full results for all four CAFA 3 evaluation procedures are shown in Additional file 1: Table S1. In terms of $F_{max}$, we obtained higher values when using FunFam domains compared to superfamilies for all three GO sub-ontologies. In fact, using superfamily annotation, DomFun performed worse than the baseline methods in most cases. Better results were found in general when predicting both GOMF and GOCC annotation than GOBP; this trend also occurs with the top CAFA 3 methods [22].

**Table 1:** Maximum *F*-measure ($F_{max}$) scores obtained with DomFun using the CAFA 3 prediction benchmark

Domain classification	FA	Association + combination methods			
		HyI + Fis	PCC+Sto	Jac + Sto	Sim+Sto
$F_{max}$ values from PR curves (domain-FunFys associations for CAFA 3 target proteins)					
FunFams	GOMF	0.608	0.553	0.515	**0.624**
	GOBP	0.452	0.444	0.443	**0.492**
	GOCC	0.542	0.529	0.529	**0.602**
Superfamilies	GOMF	0.314	0.347	0.350	**0.384**
	GOBP	0.099	0.172	**0.238**	0.174
	GOCC	0.254	0.353	** 0.398**	0.340

The best performing methods for each domain/GO subontology combination are indicated in bold

*FA* Functional annotation, *HyI* hypergeometric index, *Sim* Simpson index, *PCC* Pearson correlation coefficient, *Jac* Jaccard index, *Sto* Stouffer’s combination method, *Fis* Fisher’s combined probability test. CAFA 3 evaluation procedure set to partial mode and limited knowledge

Simpson index with Stouffer’s method applied to FunFams ranked highest on average amongst all association indices implemented here using NetAnalyzer, for all evaluation procedures across all sub-ontologies, according to both $F_{max}$ and minimum semantic distance ($S_{min}$). Moreover, for $F_{max}$ it was the best performing method in all cases, with the exception of the no knowledge, full evaluation (Type 1, Mode 1) comparison for the molecular function sub-ontology (Table 2). Similar results were seen for the $S_{min}$ output measure (Additional file 2: Table S2).

**Table 2:** DomFun ranking analysis based on $F_{max}$ comparing different evaluation methods

Ontology	Type	Mode	FF-HyI-Fis	FF-PCC-Sto	FF-Jac-Sto	FF-Sim-Sto	SF-HyI-Fis	SF-PCC-Sto	SF-Jac-Sto	SF-Sim-Sto
GOMF	1	1	2.5	4.5	4.5	2.5	8	6	1	7
GOMF	1	2	2	3.5	3.5	1	8	6	5	7
GOMF	2	1	2	3	4	1	8	6	7	5
GOMF	2	2	2	3	4	1	8	7	6	5
GOBP	1	1	2	3.5	3.5	1	8	6	5	7
GOBP	1	2	2	3.5	3.5	1	8	6	5	7
GOBP	2	1	2	3.5	3.5	1	8	6	5	7
GOBP	2	2	2	3	4	1	8	6	5	7
GOCC	1	1	3	3	3	1	8	7	5	6
GOCC	1	2	2	3.5	3.5	1	8	6	5	7
GOCC	2	1	2	3.5	3.5	1	8	6	5	7
GOCC	2	2	2	3.5	3.5	1	8	6	5	7

Type 1: no knowledge, type 2: limited knowledge. Mode 1: Full, mode 2: partial. *FF* FunFams, *SF* superfamilies. *Jac* Jaccard, *Sim* Simpson, *PCC* Pearson correlation coefficient, *HyI* hypergeometric, *Sto* Stouffer, *Fis* Fisher

We compared the results obtained by DomFun to the top results from CAFA 3 for all four evaluation procedure combinations and all three GO sub-ontologies (Table 3). DomFun was the top method using GOMF for the limited knowledge partial evaluation procedure and competitive in several other situations. Similar results were found for $S_{min}$, with DomFun obtaining a lower score than the top CAFA 3 method for the no knowledge full evaluation procedure and the limited knowledge partial evaluation procedure (Additional file 3: Table S3). For both $F_{max}$ and $S_{min}$, DomFun tended to obtain worse coverage than the best performing CAFA 3 method, although this was not always the case, particularly for $S_{min}$. All values of $S_{min}$ and coverage, for all methods, sub-ontologies and validation procedures, are shown in Additional files 4 and 5: Tables S4 and S5.

**Table 3:** $F_{max}$ top values: DomFun (Simpson + Stouffer) vs. CAFA 3 methods

Ontology	Type	Mode	Top DomFun $F_{max}$	DomFun coverage	Top CAFA 3 $F_{max}$	CAFA 3 coverage
GOMF	1	1	0.357	0.71	0.618	1
GOMF	1	2	0.567	0.41	0.622	0.02
GOMF	2	1	0.431	0.49	0.622	1
GOMF	2	2	0.624	0.49	0.623	0.88
GOBP	1	1	0.275	0.46	0.397	1
GOBP	1	2	0.402	0.46	0.418	0.62
GOBP	2	1	0.37	0.55	0.598	1
GOBP	2	2	0.492	0.55	0.64	0.83
GOCC	1	1	0.412	0.49	0.615	1
GOCC	1	2	0.606	0.49	0.908	0
GOCC	2	1	0.422	0.51	0.615	1
GOCC	2	2	0.602	0.51	0.825	0

Type 1: no knowledge, type 2: limited knowledge. Mode 1: full evaluation, mode 2: partial evaluation

#### Pathway prediction performance results

We further validated DomFun using the PPP validation procedure. Precision and recall (PR) curves for are shown in the Additional files 6 and 7: Figs. S1 and S2. $F_{max}$ values are shown in Table 4. As with CAFA 3 validation, we observe better AUC-PR values for FunFam predictions than superfamilies.

**Table 4:** Maximum *F*-measure ($F_{max}$) scores for precision and recall (PR) curves obtained with DomFun using the Pathway Prediction Performance benchmark procedure

Domains classification	FA	Association + combination methods			
		HyI+Fis	PCC+Sto	Jac+Sto	Sim+Sto
$F_{max}$ values from PR curves (domains-FunFys associations from UniProt proteins)					
FunFams	GOMF	0.779	0.749	0.749	**0.850**
	GOBP	0.643	0.604	0.604	**0.714**
	GOCC	0.750	0.704	0.704	**0.824**
	KEGG	0.730	0.730	0.730	**0.822**
	Reactome	0.762	0.680	0.663	**0.822**
Superfamilies	GOMF	0.241	0.370	**0.373**	0.139
	GOBP	0.196	0.291	**0.305**	0.089
	GOCC	**0.266**	0.221	0.217	0.129
	KEGG	0.132	0.340	0.271	**0.494**
	Reactome	0.127	**0.344**	0.327	0.081

The best performing methods for each domain/annotation source  combination are indicated in bold

*FA* Functional annotation, *HyI* hypergeometric index, *PCC* Pearson correlation coefficient, *Jac* Jaccard index, *Sim* Simpson index, *Sto* Stouffer’s method, *Fis* Fisher’s method

We compared the $F_{max}$ values calculated using PPP (Table 4) against those for the CAFA 3 benchmark (Table 1).

We observed similar $F_{max}$ values for GOMF, GOCC and GOBP predictions for both CAFA 3 and PPP evaluations using FunFams. These results gives us confidence in the validity of DomFun for predicting KEGG and Reactome pathways. Interestingly, the predictions for these pathways lead to similar $F_{max}$ values to those calculated for GOMF and GOCC.

The highest $F_{max}$ values for predictions using FunFams correspond to those calculated using Simpson index with Stouffer’s method, in line with the results obtained using the CAFA 3 dataset and lending confidence to the potential use of PPP as a further validation system that can be extended beyond GO to other annotation databases.

With respect to the predictions performed with superfamilies, the $F_{max}$ values for GOMF, GOBP and GOCC were lower in comparison with FunFams, again suggesting that our methodology works better with FunFams, as also shown in the CAFA 3 results.

## Discussion

We have presented DomFun, a novel approach to predict protein function based on associations between domains and functions. The method is based on the same protein domains classification system used by the FunFHMMer method [9], evaluated in CAFA 3 under the name of Orengo-FunFams [22].

Although both methods are based on similar underlying data, DomFun differs fundamentally from Orengo-FunFams in terms of how it assigns functions to the test proteins. Orengo-FunFams first assigns FunFam domains to a test protein then, for each of these FunFams, obtains corresponding GO term annotations, which are scored based on their frequency among the seed sequences for the given FunFam. Parental terms of these GO terms are also obtained. Finally, the set of all domain-GO terms annotations for the test protein IS considered. This differs markedly from our approach, which first obtains GO association values for all domains in a given protein based on tripartite network analysis, and then combines these values to produce a single score for each predicted protein-annotation association using data-fusion methods.

Like the Orengo-FunFams method, DomFun performs particularly well for the no-knowledge partial evaluation and for GOMF. However, it performs less well in full evaluation procedures. The loss of training proteins due to the lack of CATH domains could explain this. As they will not form part of the association network, their information will not be available to make predictions and this could consequently decrease $F_{max}$ values.

More concretely, we obtained Fmax values of 0.624 (GOMF) and 0.492 (GOBP) for the no knowledge partial evaluation procedure (full details in Table 3); for the Orengo-FunFams method these were 0.623 (GOMF) and 0.64 (GOBP). We also performed a comparison between our method and the Orengo-FunFams method using the CAFA 2 training/testing dataset (data not shown), in which the Orengo-FunFams method evaluation showed Fmax values of 0.58 for GOMF, and 0.39 for GOBP when making predictions for Homo sapiens (Figures and 7C and 7L of the CAFA 2 supplementary material [26]). These values were similar to those obtained with DomFun (Table 1) for GOMF calculated with Simpson index with Stouffer’s method (0.592) and slightly higher for GOBP calculated with the same method (0.341).

Regarding coverage, we tended to obtain lower values than many of the top-performing CAFA 3 methods, particularly for FunFam predictions, although there were some exceptions, as shown in Table 3 and Additional file 3: Table S3. Again, we are currently limited to predicting for proteins for which domain annotation is available. However, the methodology presented here can easily be adapted to incorporate other protein features, such as amino acid k-mers or motifs. To this end have made all code fully available, allowing the user to analyse a tripartite network including any feature of interest. Future work could look into optimizing our system increasing the number of protein domains from other databases, such as Pfam or SCOP, or other protein features.

We obtained better coverage when the associations were calculated using superfamilies than with FunFams, which is not surprising as not all sequences in a superfamily are classified into FunFams. FunFams are only generated for groups of sequences where at least one member has experimental characterisation [23, 29]. As such they can be considered more functionally coherent than superfamilies, which is likely to account for the improved performance.

Simpson index with Stouffer’s combination method using FunFams was the best performing of all the methods implemented here for both the CAFA 3 and PPP benchmark, both for $F_{max}$ and $S_{min}$. In previous work by Clancy and Hovig, the Simpson index was used to calculate the similarity between pairs of genes mapped to a protein interaction matrix [30]. In their study, the authors explain that the similarity between two genes can vary significantly depending on the number of proteins used for its calculation. To solve this problem they used the Simpson index, which normalises the results of the similarity calculation by the node that has the minimum number of connections (see eq. 2 in the Implementation section) [30].

We have a similar problem, as there can be a large discrepancy in size between the numbers of proteins mapping to each domain and function. We suggest that by using Simpson here, we reduce this problem by normalizing to the smaller of the two.

Notably, there was much more variation in terms of performance for superfamilies. This may also be related to the network degree, this tended to be greater than for FunFams, as superfamilies tend to contain larger numbers of proteins. It should also be made clear that the homologous superfamily classification is based on domains having similar structures, but this does not necessarily mean they will have similar functions.

To validate our results using the CAFA 3 data, we incorporated the original Matlab scripts (https://github.com/yuxjiang/CAFA2) into an automated system that is able to extract testing and training data from the benchmark dataset, build the tripartite networks to calculate associations and combine them to predict function for the testing proteins such that we could perform validation using CAFA3 in a high-throughput manner. These scripts are available from https://github.com/ElenaRojano/DomFun_project and can be used by others to validate their own method, or to validate adaptations of our methods, for example by adding additional protein features to the tripartite network.

The PPP benchmark led to similar $F_{max}$ values compared to the CAFA 3 benchmark for all three GO sub-ontologies, especially for GOMF and GOCC. As such, we have confidence in the PPP procedure in terms of judging relative performance. Based on these assumptions, it would appear that DomFun has slightly greater accuracy when predicting KEGG and Reactome annotation than GO (Table 4).

The $F_{max}$ results for GOMF and GOCC tended to be better than for GOBP, in line with CAFA 3. These differences are likely due to the distinct focus of the different annotation systems. GOBP terms can be quite varied regarding the different activities of proteins they encompass: these terms refer to biological processes that can involve a large number of distinct molecular activities, enzymatic reactions and regulatory processes. On the other hand, GOMF terms tend to represent more concrete molecular activities. As such, GOBP can be considered harder to predict for [31]. We hypothesize that proteins with similar molecular functions require similar domains to perform their activities, whereas proteins involved the same biological process might utilise a much wider range of domain structures, although this will also depend on the level of specificity of the term within the GO hierarchy.

This is also commented on in the CAFA 2 manuscript, where they argue that predictors return different results depending on the type of ontology used, and that their size (number of elements), depth (maximum degree of specificity) and branching factor (number of total connections between nodes) can affect the results [26].

As mentioned above, the PPP benchmark showed better results for KEGG than GO. These resources include well-curated representations of specific metabolic pathways with shared catalytic activities performed by multiple proteins, which may explain these findings. Further work could investigate methodology to select the more specific GO terms using semantic similarity measures [32].

## Conclusion

Results for both CAFA 3 and PPP validation show that, of the 8 prediction frameworks implemented here, combining the four association metrics with the two protein domain annotation types (superfamilies and FunFam), the best combination for making domain-function predictions with DomFun is the Simpson index with Stouffer’s method using FunFams.

We have shown that protein domain associations based on network analysis can be useful for predicting protein function for multiple species, showing comparable performance when predicting GO annotation to other methods based on structural domain-based in the CAFA 3 challenge. We have also presented a novel validation system for protein function prediction that shows similar results to the CAFA 3 benchmark, but can also be extended to use KEGG and Reactome annotation.

DomFun has been implemented in such a way that other protein features, such as different domain annotation classifications can also be integrated. Future work of DomFun should focus on improving domain-function associations using additional protein-features for protein annotation, more domain annotations and predicting for a greater range of functional systems.

### Availability and requirements

Project name: DomFun Project home page: https://github.com/ElenaRojano/DomFun_project Operating system(s): Unix-like systems Programming language: Ruby, Matlab Other requirements: CAFA 3 benchmarking system used to compare methods License: GNU GPL Any restrictions to use by non-academics: None.

## Supplementary information

**Additional file 1. Table S1**: *F*_*max*_ values for all DomFun methods and all combinations of evaluation scenarios and ontologies, compared to the highest equivalent value from CAFA 3 and the baseline methods. Type 1: no knowledge, type 2: limited knowledge. Mode 1: Full, mode 2: partial. FF: FunFams, SF: superfamilies. Jac: Jaccard, Sim: Simpson, PCC: Pearson correlation coe_cient, HyI: hypergeometric. Sto: Stou_er, Fis: Fisher.

**Additional file 2. Table S2**:

**Additional file 3. Table S3**: DomFun *S*_*min*_ Top vs. CAFA *S*_*min*_ Top (top means lowest score). Type 1: no knowledge, type 2: limited knowledge. Mode 1: Full, mode 2: partial.

**Additional file 4. Table S4**: Coverage values for all DomFun methods and all combinations of evaluation scenarios and ontologies, compared to the highest equivalent value from CAFA 3 and the baseline methods. Type 1: no knowledge, type 2: limited knowledge. Mode 1: Full, mode 2: partial. FF: FunFams, SF: superfamilies. Jac: Jaccard, Sim: Simpson, PCC: Pearson correlation coefficient, HyI: hypergeometric. Sto: Stouffer, Fis: Fisher.

**Additional file 5. Table S5**: *S*_*min*_ values for all DomFun methods and all combinations of evaluation scenarios and ontologies, compared to the highest equivalent value from CAFA 3 and the baseline methods. Type 1: no knowledge, type 2: limited knowledge. Mode 1: Full, mode 2: partial. FF: FunFams, SF: superfamilies. Jac: Jaccard, Sim: Simpson, PCC: Pearson correlation coefficient, HyI: hypergeometric. Sto: Stouffer, Fis: Fisher.

**Additional file 6. Fig. S1**: Precision and recall curves to ascertain DomFun accuracy using domain-function associations with FunFams (PPP). funfamsPPP.pdf Prediction results for Gene Ontology (GO) molecular functions (GOMF, red curves), biological process (GOBP, blue curves), GOCC (green curves), KEGG (orange curves) and Reactome pathways (black curves) are shown. These curves compare DomFun results using associations between FunFam domains and functions calculated with (a) Jaccard index, (b) Pearson Correlation Coefficient (PCC), (c) hypergeometric index (HyI) and (d) Simpson index. The area under the precision-recall curve (AUC-PR) for each comparison is also shown.

**Additional file 7. Fig. S2**: Precision and recall curves to ascertain DomFun accuracy using domain-function associations with superfamilies (PPP). superfamilyPPP.pdf Prediction results for Gene Ontology (GO) molecular functions (GOMF, red curves), biological process (GOBP, blue curves), GOCC (green curves), KEGG (orange curves) and Reactome pathways (black curves) are shown. These curves compare DomFun results using associations between superfamily domains and functions calculated with (a) Jaccard index, (b) Pearson Correlation Coefficient (PCC), (c) hypergeometric index (HyI) and (d) Simpson index. The area under the precision-recall curve (AUC-PR) for each comparison is also shown.

## Data Availability

The source code and datasets used in this research can be downloaded from https://rubygems.org/gems/DomFun, https://github.com/ElenaRojano/DomFun and https://github.com/ElenaRojano/DomFun_project.

## Abbreviations

AUC-PR: Area under the curve-precision and recall
CAFA: Critical Assessment of Function Annotation
$$F_{max}$$: Maximum F-measure
FunFam: Functional family
HyI: Hypergeometric index
GO: Gene Ontology
GOBP: Gene Ontology biological process
GOCC: Gene Ontology cellular component
GOMF: Gene Ontology molecular function
KEGG: Kyoto Encyclopedia of Genes and Genomes
PCC: Pearson correlation coefficient
PR: Precision and recall
$S_{min}$: Minimum semantic difference
