# Supplementary material for: Assigning protein function from domain-function associations using DomFun
Source: BMC Bioinformatics. 2022 Jan 15;23:43. doi: 10.1186/s12859-022-04565-6 (PMC8761305; doi:10.1186/s12859-022-04565-6)
Supplement: Supplementary file 1 — Additional file 1. Table S1: Fmax values for all DomFun methods and all combinations of evaluation scenarios and ontologies, compared to the highest equivalent value from CAFA 3 and the baseline methods. Type 1: no knowledge, type 2: limited knowledge. Mode 1: Full, mode 2: partial. FF: FunFams, SF: superfamilies. Jac: Jaccard, Sim: Simpson, PCC: Pearson correlation coe_cient, HyI: hypergeometric. Sto: Stou_er, Fis: Fisher. [file 12859_2022_4565_MOESM1_ESM.pdf]

Table 1: F_{max} values for all DomFun methods and all combinations of evaluation scenarios and ontologies, compared to the highest equivalent value from CAFA 3 and the baseline methods. Type 1: no knowledge, type 2: limited knowledge. Mode 1: Full, mode 2: partial. FF: FunFams, SF: superfamilies. Jac: Jaccard, Sim: Simpson, PCC: Pearson correlation coefficient, HyI: hypergeometric. Sto: Stouffer, Fis: Fisher.

Ontology	Type	Mode	FF-HyI-Fis	FF-PCC-Sto	FF-Jac-Sto	FF-Sim-Sto	SF-HyI-Fis	SF-PCC-Sto	SF-Jac-Sto	SF-Sim-Sto	Top CAFA 3	BLAST	Naïve
GOMF	1	1	0.346	0.345	0.345	0.346	0.191	0.338	0.357	0.264	0.618	0.254	0.344
GOMF	1	2	0.553	0.547	0.547	0.567	0.2	0.385	0.416	0.297	0.622	0.257	0.344
GOMF	2	1	0.429	0.39	0.381	0.431	0.293	0.319	0.304	0.35	0.622	0.363	0.299
GOMF	2	2	0.608	0.553	0.515	0.624	0.314	0.347	0.35	0.384	0.623	0.368	0.299
GOBP	1	1	0.27	0.269	0.269	0.275	0.102	0.158	0.191	0.14	0.397	0.198	0.242
GOBP	1	2	0.374	0.361	0.361	0.402	0.106	0.169	0.209	0.15	0.418	0.198	0.242
GOBP	2	1	0.358	0.353	0.353	0.37	0.097	0.153	0.203	0.123	0.598	0.463	0.443
GOBP	2	2	0.452	0.444	0.443	0.492	0.099	0.157	0.209	0.125	0.64	0.464	0.443
GOCC	1	1	0.403	0.403	0.403	0.412	0.26	0.329	0.352	0.33	0.615	0.434	0.553
GOCC	1	2	0.568	0.566	0.566	0.606	0.28	0.372	0.406	0.368	0.908	0.447	0.553
GOCC	2	1	0.403	0.398	0.398	0.422	0.24	0.319	0.354	0.31	0.615	0.439	0.52
GOCC	2	2	0.542	0.529	0.529	0.602	0.254	0.353	0.398	0.34	0.825	0.454	0.52
