# Supplementary material for: Assigning protein function from domain-function associations using DomFun
Source: BMC Bioinformatics. 2022 Jan 15;23:43. doi: 10.1186/s12859-022-04565-6 (PMC8761305; doi:10.1186/s12859-022-04565-6)
Supplement: Supplementary file 2 — Additional file 2. Table S2: [file 12859_2022_4565_MOESM2_ESM.pdf]

Table 2: Ranking of different DomFun methods according to S_{min}. Type 1: no knowledge, type 2: limited knowledge. Mode 1: Full, mode 2: partial. FF: FunFams, SF: superfamilies. Jac: Jaccard, Sim: Simpson, PCC: Pearson correlation coefficient, HyI: hypergeometric. Sto: Stouffer, Fis: Fisher.

Ontology	Type	Mode	FF-HyI-Fis	FF-PCC-Sto	FF-Jac-Sto	FF-Sim-Sto	SF-HyI-Fis	SF-PCC-Sto	SF-Jac-Sto	SF-Sim-Sto
GOMF	1	1	2	3	4	1	8	7	6	5
GOMF	1	2	4	2	3	1	8	7	6	5
GOMF	2	1	3	2	4	1	8	7	6	5
GOMF	2	2	5	2	3	1	8	7	6	4
GOBP	1	1	4	2	3	1	8	6	5	7
GOBP	1	2	4	2	1	3	8	6	5	7
GOBP	2	1	4	2	3	1	8	6	5	7
GOBP	2	2	4	1	2	3	8	6	5	7
GOCC	1	1	3	2	4	1	8	6	5	7
GOCC	1	2	4	2	3	1	8	6	5	7
GOCC	2	1	4	2	3	1	8	6	5	7
GOCC	2	2	4	2	3	1	8	6	5	7
