# Supplementary material for: Assigning protein function from domain-function associations using DomFun
Source: BMC Bioinformatics. 2022 Jan 15;23:43. doi: 10.1186/s12859-022-04565-6 (PMC8761305; doi:10.1186/s12859-022-04565-6)
Supplement: Supplementary file 3 — Additional file 3. Table S3: DomFun Smin Top vs. CAFA Smin Top (top means lowest score). Type 1: no knowledge, type 2: limited knowledge. Mode 1: Full, mode 2: partial. [file 12859_2022_4565_MOESM3_ESM.pdf]

Table 3: DomFun S_{min} Top vs. CAFA S_{min} Top (top means lowest score). Type 1: no knowledge, type 2: limited knowledge. Mode 1: Full, mode 2: partial.

Ontology	Type	Mode	DomFun Top S_{min}	Domfun Top coverage	CAFA 3 Top S_{min}	CAFA 3 Top coverage
GOMF	1	1	6.185	0.41	6.262	1
GOMF	1	2	5.39	0.41	4.302	0.02
GOMF	2	1	4.965	0.49	4.894	1
GOMF	2	2	4.326	0.49	4.894	1
GOBP	1	1	16.01	0.46	14.784	1
GOBP	1	2	15.368	0.46	9.43	0.11
GOBP	2	1	10.574	0.55	8.92	1
GOBP	2	2	10.613	0.55	5.731	0
GOCC	1	1	5.566	0.49	5.121	0.88
GOCC	1	2	4.665	0.49	2.672	0.03
GOCC	2	1	5.585	0.51	5.039	1
GOCC	2	2	5.358	0.51	1.362	0
