# Supplementary material for: Assigning protein function from domain-function associations using DomFun
Source: BMC Bioinformatics. 2022 Jan 15;23:43. doi: 10.1186/s12859-022-04565-6 (PMC8761305; doi:10.1186/s12859-022-04565-6)
Supplement: Supplementary file 4 — Additional file 4. Table S4: Coverage values for all DomFun methods and all combinations of evaluation scenarios and ontologies, compared to the highest equivalent value from CAFA 3 and the baseline methods. Type 1: no knowledge, type 2: limited knowledge. Mode 1: Full, mode 2: partial. FF: FunFams, SF: superfamilies. Jac: Jaccard, Sim: Simpson, PCC: Pearson correlation coefficient, HyI: hypergeometric. Sto: Stouffer, Fis: Fisher. [file 12859_2022_4565_MOESM4_ESM.pdf]

Table 4: Coverage values for all DomFun methods and all combinations of evaluation scenarios and ontologies, compared to the highest equivalent value from CAFA 3 and the baseline methods. Type 1: no knowledge, type 2: limited knowledge. Mode 1: Full, mode 2: partial. FF: FunFams, SF: superfamilies. Jac: Jaccard, Sim: Simpson, PCC: Pearson correlation coefficient, HyI: hypergeometric. Sto: Stouffer, Fis: Fisher.

Ontology	Type	Mode	FF-HyI-Fis	FF-PCC-Sto	FF-Jac-Sto	FF-Sim-Sto	SF-HyI-Fis	SF-PCC-Sto	SF-Jac-Sto	SF-Sim-Sto	Top CAFA3	BLAST	Naïve
GOMF	1	1	0.41	0.41	0.41	0.41	0.71	0.71	0.71	0.71	1	0.96	1
GOMF	1	2	0.41	0.41	0.41	0.41	0.71	0.71	0.71	0.71	0.02	0.96	1
GOMF	2	1	0.49	0.49	0.49	0.49	0.74	0.74	0.74	0.74	1	0.98	1
GOMF	2	2	0.49	0.49	0.49	0.49	0.74	0.74	0.74	0.74	0.88	0.98	1
GOBP	1	1	0.46	0.46	0.46	0.46	0.65	0.65	0.65	0.65	1	1	1
GOBP	1	2	0.46	0.46	0.46	0.46	0.65	0.65	0.65	0.65	0.62	1	1
GOBP	2	1	0.55	0.55	0.55	0.55	0.82	0.82	0.82	0.82	1	1	1
GOBP	2	2	0.55	0.55	0.55	0.55	0.82	0.82	0.82	0.82	0.83	1	1
GOCC	1	1	0.49	0.49	0.49	0.49	0.68	0.68	0.68	0.68	1	0.93	1
GOCC	1	2	0.49	0.49	0.49	0.49	0.68	0.68	0.68	0.68	0	0.93	1
GOCC	2	1	0.51	0.51	0.51	0.51	0.72	0.72	0.72	0.72	1	0.91	1
GOCC	2	2	0.51	0.51	0.51	0.51	0.72	0.72	0.72	0.72	0	0.91	1
