# Supplementary material for: Assigning protein function from domain-function associations using DomFun
Source: BMC Bioinformatics. 2022 Jan 15;23:43. doi: 10.1186/s12859-022-04565-6 (PMC8761305; doi:10.1186/s12859-022-04565-6)
Supplement: Supplementary file 5 — Additional file 5. Table S5: Smin values for all DomFun methods and all combinations of evaluation scenarios and ontologies, compared to the highest equivalent value from CAFA 3 and the baseline methods. Type 1: no knowledge, type 2: limited knowledge. Mode 1: Full, mode 2: partial. FF: FunFams, SF: superfamilies. Jac: Jaccard, Sim: Simpson, PCC: Pearson correlation coefficient, HyI: hypergeometric. Sto: Stouffer, Fis: Fisher. [file 12859_2022_4565_MOESM5_ESM.pdf]

Table 5: S_{min} values for all DomFun methods and all combinations of evaluation scenarios and ontologies, compared to the highest equivalent value from CAFA 3 and the baseline methods. Type 1: no knowledge, type 2: limited knowledge. Mode 1: Full, mode 2: partial. FF: FunFams, SF: superfamilies. Jac: Jaccard, Sim: Simpson, PCC: Pearson correlation coefficient, HyI: hypergeometric. Sto: Stouffer, Fis: Fisher.

Ontology	Type	Mode	FF-HyI-Fis	FF-PCC-Sto	FF-Jac-Sto	FF-Sim-Sto	SF-HyI-Fis	SF-PCC-Sto	SF-Jac-Sto	SF-Sim-Sto	Top CAFA3	BLAST	Naïve
GOMF	1	1	6.453	6.503	6.631	6.185	50.62	10.006	7.895	7.192	6.262	7.227	6.775
GOMF	1	2	7.231	5.992	6.105	5.39	70.813	12.927	9.592	7.384	4.302	7.191	6.775
GOMF	2	1	5.41	5.346	5.469	4.965	40.488	8.596	7.146	5.923	4.894	5.482	5.626
GOMF	2	2	6.9	4.947	5.055	4.326	54.822	10.829	8.683	5.864	4.894	5.445	5.626
GOBP	1	1	24.242	16.097	16.141	16.01	217.253	77.798	52.58	156.669	14.784	16.908	15.893
GOBP	1	2	44.593	15.557	15.368	16.827	333.899	118.695	79.226	240.488	9.43	16.857	15.893
GOBP	2	1	19.594	10.841	10.928	10.574	342.937	109.207	69.301	269.112	8.92	10.224	9.714
GOBP	2	2	32.71	10.613	10.746	10.76	417.685	132.895	84.191	327.729	5.731	10.225	9.714
GOCC	1	1	5.91	5.88	6.066	5.566	40.632	11.753	9.515	19.722	5.121	6.349	5.456
GOCC	1	2	6.252	5.275	5.564	4.665	59.348	16.173	12.554	28.244	2.672	6.328	5.456
GOCC	2	1	6.721	5.936	5.992	5.585	47.43	12.61	10.195	24.806	5.039	6.119	5.621
GOCC	2	2	9.51	5.942	5.972	5.358	65.712	16.771	13.203	34.076	1.362	6.051	5.621
