# Supplementary figures and images for: Assigning protein function from domain-function associations using DomFun
Source: BMC Bioinformatics. 2022 Jan 15;23:43. doi: 10.1186/s12859-022-04565-6 (PMC8761305; doi:10.1186/s12859-022-04565-6)

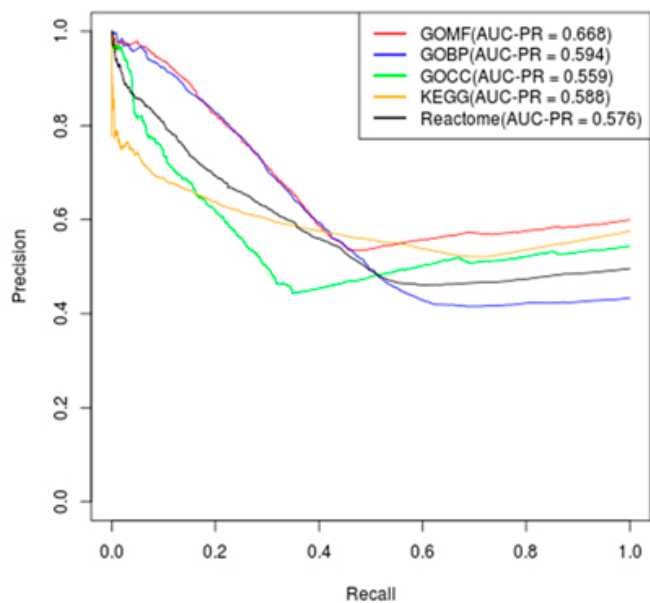

(a) Jaccard

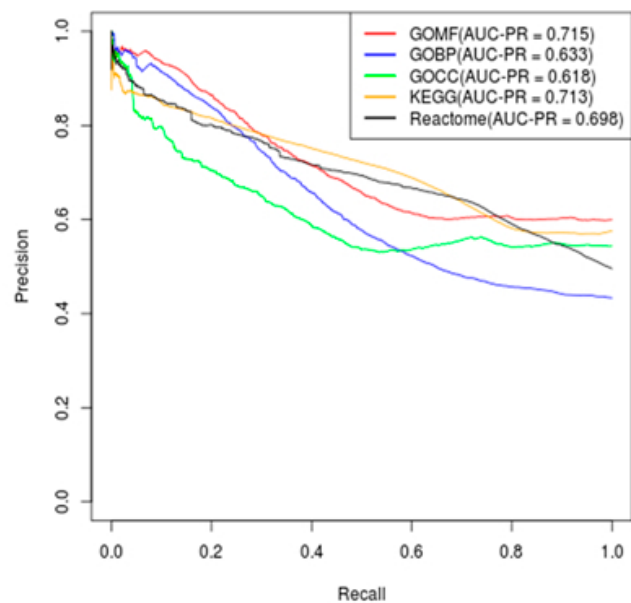

(b) PCC

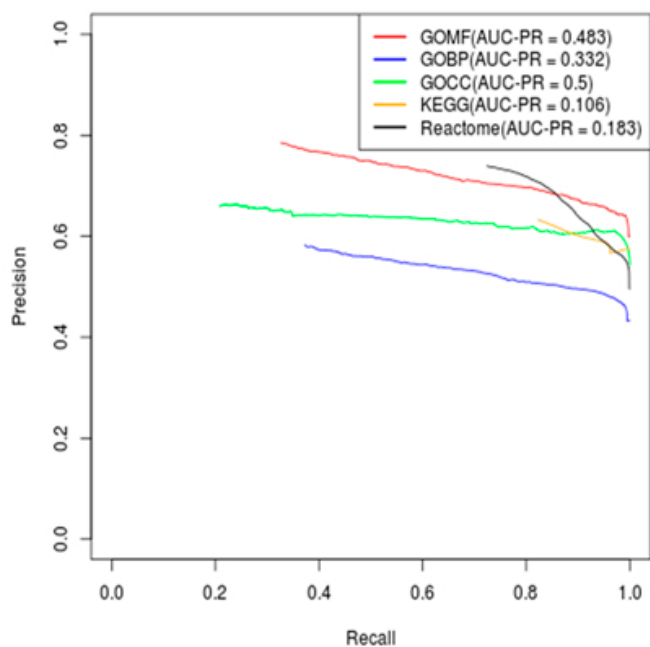

(c) Hyl

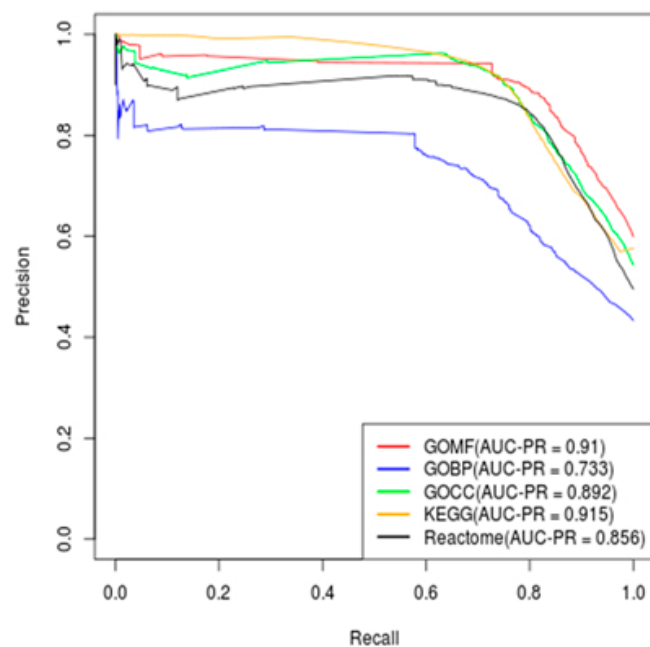

(d) Simpson

Supplement: Supplementary file 6 — Additional file 6. Fig. S1: Precision and recall curves to ascertain DomFun accuracy using domain-function associations with FunFams (PPP). funfamsPPP.pdf Prediction results for Gene Ontology (GO) molecular functions (GOMF, red curves), biological process (GOBP, blue curves), GOCC (green curves), KEGG (orange curves) and Reactome pathways (black curves) are shown. These curves compare DomFun results using associations between FunFam domains and functions calculated with (a) Jaccard index, (b) Pearson Correlation Coefficient (PCC), (c) hypergeometric index (HyI) and (d) Simpson index. The area under the precision-recall curve (AUC-PR) for each comparison is also shown. [file 12859_2022_4565_MOESM6_ESM.pdf]

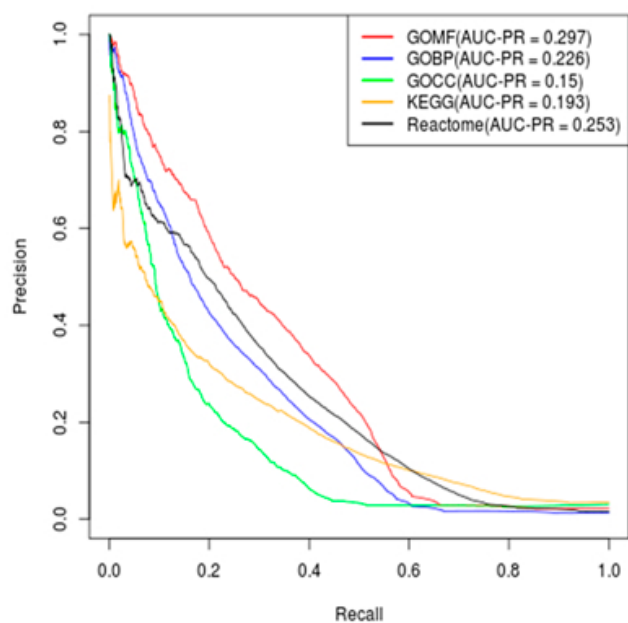

(a) Jaccard

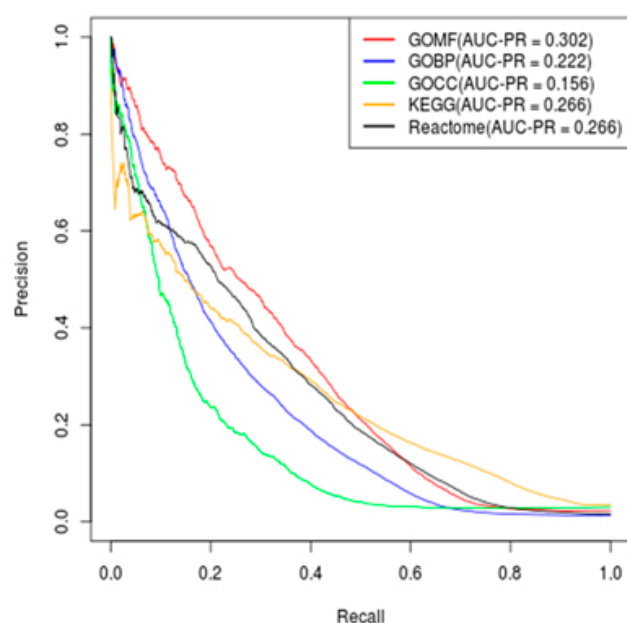

(b) PCC

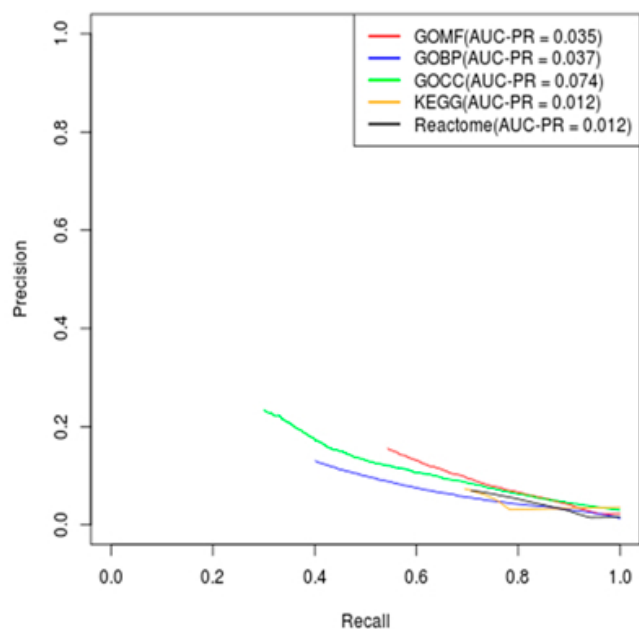

(c) Hyl

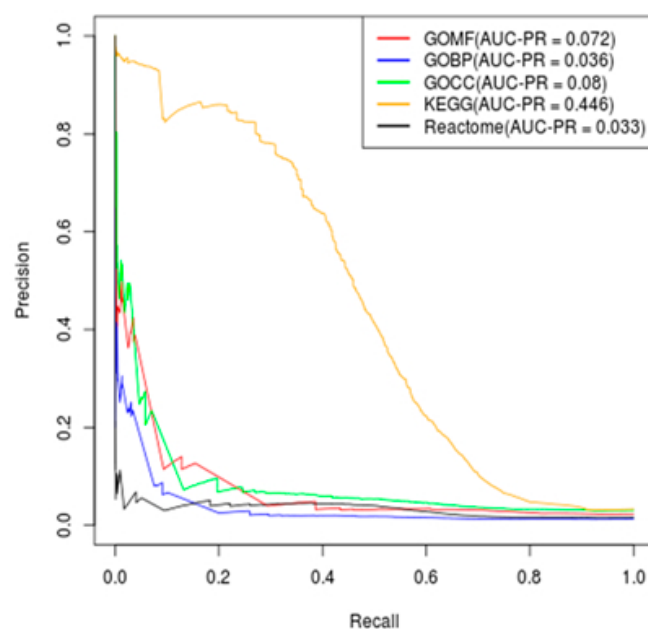

(d) Simpson

Supplement: Supplementary file 7 — Additional file 7. Fig. S2: Precision and recall curves to ascertain DomFun accuracy using domain-function associations with superfamilies (PPP). superfamilyPPP.pdf Prediction results for Gene Ontology (GO) molecular functions (GOMF, red curves), biological process (GOBP, blue curves), GOCC (green curves), KEGG (orange curves) and Reactome pathways (black curves) are shown. These curves compare DomFun results using associations between superfamily domains and functions calculated with (a) Jaccard index, (b) Pearson Correlation Coefficient (PCC), (c) hypergeometric index (HyI) and (d) Simpson index. The area under the precision-recall curve (AUC-PR) for each comparison is also shown. [file 12859_2022_4565_MOESM7_ESM.pdf]
